# Undergraduate academic assessment and dental licensing examination outcomes: a single-institution retrospective cohort study in China

**DOI:** 10.1186/s12909-026-09971-5

**Published:** 2026-07-18

**Authors:** Yiqi Wang, Siqi Xia, Luobing Yu, Shixuan Wang, Ming Zhang, Biyao Li, Xiang Wang, Mingyuan Peng, Yuan Yuan, Michael George Botelho, Jinjing Guo, Shuhua Wang

**Affiliations:** 1 School of Stomatology, Zhejiang Chinese Medical University, Hangzhou, Zhejiang 310053 China; 2 Stomatology Hospital Affiliated to Zhejiang Chinese Medical University, Hangzhou, Zhejiang 310053 China; 3 Faculty of Dentistry, The University of Hong Kong, Hong Kong SAR, China

**Keywords:** Dental licensing qualification examination, Undergraduate dental education, Curriculum–assessment alignment, Academic assessment, Phase-based examination, Licensing outcomes, Practical examination

## Abstract

**Background:**

Licensing examinations provide an external reference for selected knowledge, skills, and professional standards required for entry into clinical practice. In China, the Dental Licensing Qualification Examination (DLQE) marks the transition from undergraduate dental education to licensed practice. This study examined associations between undergraduate academic assessment indicators and DLQE outcomes to inform curriculum–assessment alignment and longitudinal academic monitoring.

**Methods:**

This retrospective cohort study included graduates from Zhejiang Chinese Medical University between 2019 and 2023. Of 315 graduates, 278 had available DLQE outcomes and were included in the overall analysed cohort. Practical-score analyses included all 278 graduates, whereas written-score and cognitive-domain analyses included 276 graduates because two graduates were not eligible for the written examination after not passing the practical examination. Undergraduate course scores were grouped into five predefined credit-weighted course clusters mapped to DLQE written subject areas. Basic Comprehensive Examination (BCE) and Professional Comprehensive Examination (PCE) scores were examined as complementary phase-based indicators among graduates from the 2021–2023 cohorts with complete data (*n* = 172). Multivariable linear regression models included graduation cohort as a fixed factor. Internship institution type was examined as a broad proxy for clinical training context.

**Results:**

Dental clinical course performance showed the most consistent associations with written DLQE scores and written cognitive-domain scores. Basic science course performance showed weaker associations in the primary models, but these were not retained in sensitivity analyses restricted to the 2021–2023 cohorts. Associations with practical DLQE scores were weaker and less consistent. BCE and PCE were associated with written DLQE scores and all three written cognitive-domain scores; BCE showed a weak association with practical scores, whereas PCE was not associated with practical scores. Internship institution type showed no clear independent association with DLQE outcomes after adjustment.

**Conclusions:**

Within this single-institution cohort, undergraduate academic indicators were more consistently associated with written and cognitive-domain DLQE outcomes than with practical examination performance. DLQE outcomes may provide useful but incomplete external reference points for curriculum monitoring and assessment alignment in undergraduate dental education.

**Supplementary Information:**

The online version contains supplementary material available at 10.1186/s12909-026-09971-5.

## Background

Licensing examinations are used in health professions education to assess selected knowledge, skills, and professional standards required for entry into clinical practice. They also provide an external reference point for interpreting how undergraduate curricula and assessment systems relate to professional licensure expectations [1–3]. For dental education, this relationship is particularly important because undergraduate programmes must prepare students to integrate biomedical knowledge, dental knowledge, clinical reasoning, and patient care [3, 4].

In China, the Dental Licensing Qualification Examination (DLQE) is the national licensure examination for dental graduates and marks the transition from undergraduate dental education (UDE) to licensed clinical practice. Under the Physicians Law of the People’s Republic of China and the Interim Measures for the Physician Qualification Examination, dental graduates are eligible to take the DLQE after completing one year of supervised clinical practice [5, 6]. The DLQE consists of a practical examination followed by a medical comprehensive written examination. Candidates must pass the practical examination before taking the written examination, and successful completion of both stages is required to obtain physician qualification in China [6, 7].

The practical examination assesses oral examination skills, disease prevention and treatment procedures, emergency skills, history taking, and case analysis. The written examination covers medical humanities, basic sciences, general clinical medicine, preventive medicine, and dental clinical disciplines [7].

Official DLQE performance reports also classify written examination performance into three cognitive domains: memory, understanding, and application. These are not separate examinations, but analytical categories based on the cognitive level of written test items. Memory refers to recall of factual and conceptual knowledge. Understanding refers to comprehension, interpretation, and explanation of knowledge. Application refers to using knowledge to solve clinical or problem-based tasks. These cognitive-domain scores should be interpreted as subdomains of written test performance rather than direct measures of clinical performance. They allow written DLQE performance to be examined beyond total scores and help evaluate whether undergraduate academic indicators are differentially associated with recall-, interpretation-, and application-oriented written performance.

UDE in China usually follows a progressive five-year curriculum, moving from foundational biomedical sciences to general clinical medicine, discipline-specific dental courses, and clinical practice. Medical humanities and preventive medicine are also included as components of professional and public health education [4]. This curricular structure appears to overlap with the subject areas represented in the DLQE written examination. Course-cluster performance may therefore provide a more domain-specific view of undergraduate academic assessment than a single overall grade.

In addition to course-based assessment, phase-based examinations may provide information about students’ academic development across training stages. At Zhejiang Chinese Medical University (ZCMU), the Basic Comprehensive Examination (BCE) focuses on foundational biomedical knowledge, whereas the Professional Comprehensive Examination (PCE) focuses on integrated dental professional knowledge and clinically relevant reasoning. These examinations were introduced as institutional phase-based assessments to support staged academic monitoring as students progressed from theoretical and discipline-based learning toward integrated professional knowledge and preparation for licensing assessment.

Clinical internship is also an essential component of the five-year dental curriculum. It provides supervised exposure to clinical environments where students consolidate knowledge, observe patient care, and develop procedural skills. Because clinical learning may vary with supervision, feedback, case exposure, procedural opportunities, and workplace-based learning, internship context may be relevant when examining how undergraduate education relates to later licensing outcomes [8, 9].

This study was informed by constructive alignment and competence development theory. Constructive alignment emphasises coherence among learning outcomes, teaching activities, and assessment methods [10]. Competence development theory further suggests that professional competence develops progressively through foundational knowledge, clinical exposure, and integrated reasoning [11]. In this study, these theories were used as interpretive frameworks for examining associations between undergraduate assessment and later licensing outcomes, rather than as directly tested theoretical models.

Previous studies in medical and dental education have shown that earlier academic performance is associated with later academic or licensure-related outcomes [3, 12]. However, less is known about how different undergraduate academic indicators are associated with distinct dimensions of licensure performance. This distinction is important because written, cognitive-domain, and practical scores reflect different assessment formats and selected competence-related domains [8, 9, 13, 14].

Using DLQE outcomes as an external licensure reference, this study examined associations between undergraduate academic assessment indicators and later DLQE outcomes among graduates of a five-year stomatology programme. We examined course-cluster performance and phase-based examinations as undergraduate academic assessment indicators, and considered internship institution type as a contextual factor related to clinical training. By identifying areas of stronger and weaker association, this study may inform interpretation of curriculum–assessment alignment and longitudinal academic monitoring in undergraduate dental education.

## Methods

### Study design and participants

This retrospective cohort study was conducted at the School of Stomatology, ZCMU, China. The source population comprised 315 graduates from the five-year stomatology programme between 2019 and 2023. Among them, 278 had available DLQE outcomes and were included in the overall analysed cohort; 37 were excluded because DLQE outcomes were unavailable. No graduate was excluded because of failure to obtain a degree.

Because the DLQE is staged, practical-score analyses and first-attempt pass-rate calculations included all 278 graduates, whereas written-score and cognitive-domain analyses included 276 graduates. Two graduates did not pass the practical examination and were not eligible for the written examination; their written and cognitive-domain outcomes were treated as not applicable rather than as zero.

BCE and PCE analyses were treated as secondary analyses and were restricted to graduates with available BCE, PCE, graduation cohort, and DLQE outcome data. These examinations were introduced beginning with the 2021 graduating cohort as institutional phase-based assessments for staged academic monitoring; therefore, BCE and PCE data were available only for the 2021–2023 cohorts (*n* = 172). Characteristics of the overall analysed cohort and the 2021–2023 restricted cohort are provided in Supplementary Table S1.

### Data sources and variables

Data were extracted from de-identified institutional records maintained by the Academic Affairs Office of ZCMU. The dataset included undergraduate course-cluster scores, BCE and PCE scores, graduation cohort, internship institution type, postgraduation variables, and DLQE outcomes.

DLQE outcomes included first-attempt pass status, practical examination scores, written examination scores, and cognitive-domain scores within the written examination. According to DLQE regulations, candidates must pass the practical examination before being eligible for the written examination [7]. The practical and written examinations have maximum scores of 100 and 600, with passing thresholds of 60 and 360, respectively. Cognitive-domain scores were individual-level scores derived from official DLQE written examination performance reports and were classified into memory, understanding, and application domains. These scores were interpreted as written subdomain scores rather than direct measures of clinical performance.

### Undergraduate academic indicators

Undergraduate academic indicators included course-cluster scores and phase-based examination scores. Course-cluster scores were used as the primary undergraduate academic indicators. Five predefined course clusters were used: Basic science, Dental clinical, General clinical, Medical humanities, and Preventive medicine. Individual course scores were reported on a 100-point scale and reflected both continuous assessment and final examination performance. Course assessments followed institutional academic affairs requirements and course syllabi, including predefined assessment components, item-type proportions, approximate difficulty distribution, and review of examination papers and final grades. For the cohorts included in this study, the assessment framework for the same course was generally maintained across cohorts and delivered by stable teaching teams; however, formal cross-course measurement standardisation and reliability indices were not available for the present analysis. Course-cluster scores were calculated as credit-weighted mean final course scores using the formula Σ(course final score × course credit) / Σ(course credit).

Course clustering and mapping were established before analysis according to the national DLQE framework and the undergraduate curriculum structure, rather than according to statistical results. Of the 22 DLQE written subjects, 20 had direct counterparts in the undergraduate curriculum and were included in the course-level mapping. Gynecology and pediatrics were not mapped because they were offered as elective clinical courses with very low enrolment and did not have comparable course-grade data for cohort-level analysis. The mapping between undergraduate course clusters and DLQE written subjects is provided in Supplementary Table S2.

Phase-based examination scores were examined as complementary undergraduate academic indicators. BCE was administered in the third year and assessed foundational biomedical knowledge, whereas PCE was administered in the fourth year and assessed dental and clinically relevant professional knowledge. Both were institutionally administered 100-point examinations with 60 as the passing threshold. BCE and PCE consisted of multiple-choice items selected from a standardised curriculum-aligned question bank and were developed according to institutional examination blueprints, with standardised content coverage, item-type distribution, approximate difficulty distribution, scoring procedures, and pass thresholds across implementation cohorts. Item-level response records were retained in the cloud-based marking system, but formal reliability indices and item-level quality indicators were not extracted for the present analysis.

### Secondary and exploratory variables

Internship institution type was examined as a categorical secondary institutional-level indicator of clinical training context. Institutions were categorised into four groups reflecting teaching affiliation and hospital level: Zhejiang University (ZJU) affiliated hospitals, ZCMU affiliated hospitals, Other hospitals in Hangzhou, and Other prefecture-level hospitals. This variable was used as a broad institutional proxy and did not directly measure supervision quality, case mix, clinical procedure volume, feedback, or student autonomy.

Postgraduation pathway was analysed exploratorily and reported only in the supplementary materials. It was categorised as neither employment nor postgraduate study, employment, or postgraduate study.

### Statistical analysis

Continuous variables were summarised as means and standard deviations, and categorical variables as frequencies and percentages. First-attempt pass status was used to calculate the first-attempt pass rate and was not modelled because of the limited number of failures and the study focus on continuous DLQE outcomes. Analyses were performed using SPSS version 25.0 (IBM Corp., Armonk, NY, USA), with *P* < 0.05 considered statistically significant.

Multivariable linear regression models were used to examine associations between undergraduate academic indicators and continuous DLQE outcomes. Written and practical scores were treated as primary continuous outcomes, while memory-domain, understanding-domain, and application-domain scores were treated as written cognitive-domain outcomes. Graduation cohort was included as a fixed factor. The primary course-cluster models entered the five course-cluster scores simultaneously. Course-cluster, BCE, and PCE scores were scaled so that coefficients represented the estimated change in DLQE outcome score associated with a 10-point increase in the corresponding academic indicator.

Phase-based examination models included BCE and PCE scores among graduates with available phase-based examination data. Because BCE and PCE data were available only from the 2021–2023 cohorts, sensitivity analyses of the primary course-cluster models were conducted after restricting the sample to these cohorts. Secondary adjusted models examined internship institution type after adjustment for graduation cohort and the five course-cluster scores. Exploratory analyses examined postgraduation pathway and are reported in the supplementary materials.

Model diagnostics included standardised residuals, Cook’s distance, variance inflation factors, and adjusted R². Findings were interpreted with emphasis on effect estimates, confidence intervals, and consistency across related outcomes.

## Results

### Participant characteristics

Among 315 graduates from the 2019–2023 cohorts, 278 had available DLQE outcome data and were included in the overall analysed cohort. The remaining 37 graduates were excluded because DLQE outcomes were unavailable. The analysed cohort included graduates from all five cohorts, with an overall first-attempt DLQE pass rate of 88.1%. Practical-score analyses included all 278 graduates, whereas written-score and cognitive-domain analyses included 276 graduates because two graduates were not eligible for the written examination after not passing the practical examination. Cohort characteristics, internship institution type, and key DLQE outcomes are summarised in Table 1.

For the phase-based examination analyses, 172 graduates had available BCE, PCE, graduation cohort, and DLQE outcome data. Characteristics of the overall analysed cohort and the 2021–2023 restricted cohort are provided in Supplementary Table S2. Exploratory analyses of DLQE outcomes by postgraduation pathway are provided in Supplementary Table S3.

**Table 1:** Characteristics of the analysed cohort and key DLQE outcomes

Variable group	Category	Value
Graduation cohort	2019	53 (19.1)
	2020	53 (19.1)
	2021	59 (21.2)
	2022	60 (21.6)
	2023	53 (19.1)
DLQE outcomes	First-attempt DLQE pass	245 (88.1)
	Written DLQE score	410.58 ± 40.93
	Practical DLQE score	78.06 ± 6.95
Phase-based examination	Complete BCE, PCE, and DLQE data	172 (61.9)
Internship institution type	ZJU-affiliated hospitals	124 (44.6)
	ZCMU-affiliated hospitals	96 (34.5)
	Other hospitals in Hangzhou	36 (12.9)
	Other prefecture-level hospitals	22 (7.9)

Data are presented as *n* (%) or mean ± SD. The analysed cohort comprised 278 graduates with available DLQE outcome data. Practical-score analyses and first-attempt pass-rate calculations included all 278 graduates. Written DLQE scores were calculated among 276 graduates with available written examination results. Internship institution type was classified according to internship placement records. The ZJU-affiliated hospitals category included Zhejiang University-affiliated hospitals and Sir Run Run Shaw Hospital

### Course-cluster scores and DLQE outcomes

In the primary multivariable models including the five course-cluster scores and graduation cohort, Dental clinical course performance showed the most consistent positive associations with written DLQE outcomes. A 10-point higher Dental clinical course performance score was associated with higher written DLQE scores (B = 22.495, 95% CI 9.034 to 35.956, *P* = 0.001), memory-domain scores (B = 3.063, 95% CI 0.487 to 5.639, *P* = 0.020), understanding-domain scores (B = 7.769, 95% CI 2.443 to 13.096, *P* = 0.004), and application-domain scores (B = 11.663, 95% CI 4.909 to 18.416, *P* = 0.001).

Basic science course performance showed weaker positive associations with written DLQE scores (B = 12.248, 95% CI 0.355 to 24.141, *P* = 0.044) and understanding-domain scores (B = 6.474, 95% CI 1.767 to 11.180, *P* = 0.007), but was not clearly associated with memory-domain or application-domain scores. Medical humanities course performance showed a weak positive association with practical DLQE scores in the primary model (B = 2.426, 95% CI 0.283 to 4.568, *P* = 0.027), but the estimate was small and should be interpreted cautiously. General clinical course performance and Preventive medicine course performance showed no clear independent associations with DLQE outcomes.

Overall, Dental clinical course performance showed the most consistent associations with written DLQE scores and written cognitive-domain scores. Associations with practical DLQE scores were weaker and less consistent. Results for written, practical, and cognitive-domain scores are shown in Tables 2 and 3. Standardised β coefficients are additionally reported to facilitate comparison of effect sizes across predictors.

**Table 2:** Associations between course clusters and DLQE outcomes. Panel A. Associations with total written and practical DLQE scores

Course cluster	Written score B per 10-point increase (95% CI)	Standardised β	*P*	Practical score B per 10-point increase (95% CI)	Standardised β	*P*
Basic science	12.248 (0.355 to 24.141)	0.190	0.044	-1.372 (-3.865 to 1.121)	-0.126	0.279
Dental clinical	22.495 (9.034 to 35.956)	0.297	0.001	2.228 (-0.561 to 5.016)	0.174	0.117
General clinical	3.533 (-6.201 to 13.268)	0.055	0.475	0.969 (-1.068 to 3.005)	0.088	0.350
Medical humanities	2.630 (-7.663 to 12.923)	0.046	0.615	2.426 (0.283 to 4.568)	0.246	0.027
Preventive medicine	6.460 (-2.031 to 14.950)	0.132	0.135	0.418 (-1.362 to 2.198)	0.050	0.644

Models included the five credit-weighted mean course-cluster scores simultaneously, with graduation cohort included as a fixed factor. B values indicate the estimated change in DLQE outcome score associated with a 10-point increase in the corresponding credit-weighted mean course-cluster score. Standardised β coefficients are reported to facilitate comparison of effect sizes across predictors. Practical-score analyses included all graduates with available practical examination scores (*n* = 278), whereas written-score and cognitive-domain analyses included graduates with available written examination results (*n* = 276). Written and cognitive-domain outcomes for graduates who were not eligible for the written examination were treated as not applicable

**Table 3:** Associations between course clusters and DLQE outcomes. Panel B. Associations with cognitive-domain scores within the written DLQE

Course cluster	Memory-domain B per 10-point increase (95% CI)	Standardised β	*P*	Understanding-domain B per 10-point increase (95% CI)	Standardised β	*P*	Application-domain B per 10-point increase (95% CI)	Standardised β	*P*
Basic science	1.960 (-0.315 to 4.236)	0.069	0.091	6.474 (1.767 to 11.180)	0.199	0.007	3.814 (-2.152 to 9.781)	0.106	0.209
Dental clinical	3.063 (0.487 to 5.639)	0.092	0.020	7.769 (2.443 to 13.096)	0.203	0.004	11.663 (4.909 to 18.416)	0.275	0.001
General clinical	-0.009 (-1.872 to 1.854)	0.000	0.993	0.805 (-3.047 to 4.658)	0.025	0.681	2.737 (-2.147 to 7.620)	0.076	0.271
Medical humanities	1.517 (-0.452 to 3.487)	0.060	0.131	-0.099 (-4.172 to 3.975)	-0.003	0.962	1.211 (-3.953 to 6.375)	0.037	0.645
Preventive medicine	0.933 (-0.691 to 2.558)	0.043	0.259	2.907 (-0.453 to 6.267)	0.117	0.090	2.619 (-1.640 to 6.879)	0.095	0.227

Models included the five credit-weighted mean course-cluster scores simultaneously, with graduation cohort included as a fixed factor. B values indicate the estimated change in DLQE outcome score associated with a 10-point increase in the corresponding credit-weighted mean course-cluster score. Standardised β coefficients are reported to facilitate comparison of effect sizes across predictors. Practical-score analyses included all graduates with available practical examination scores (*n* = 278), whereas written-score and cognitive-domain analyses included graduates with available written examination results (*n* = 276). Written and cognitive-domain outcomes for graduates who were not eligible for the written examination were treated as not applicable

In sensitivity analyses restricted to the 2021–2023 cohorts, Dental clinical course performance remained associated with written DLQE scores (B = 33.623, 95% CI 16.672 to 50.575, *P* < 0.001) and all written cognitive-domain scores. Associations observed for Basic science course performance in the primary models were not retained. Medical humanities course performance showed a weak association with practical DLQE scores, but this finding was interpreted cautiously. Detailed results are provided in Supplementary Table S4.

### Phase-based examinations and DLQE outcomes

Among the 172 graduates with available phase-based examination data, multivariable models showed that both BCE and PCE were associated with higher written DLQE scores. A 10-point increase in BCE score was associated with a 14.355-point increase in written DLQE score (95% CI 4.105 to 24.605, *P* = 0.006), while a 10-point increase in PCE score was associated with an 18.455-point increase in written DLQE score (95% CI 9.612 to 27.299, *P* < 0.001).

Both phase-based examinations were also associated with memory-domain, understanding-domain, and application-domain scores within the written DLQE. For memory-domain scores, BCE and PCE both showed positive associations. For understanding-domain and application-domain scores, both examinations were also positively associated with the outcomes, with PCE showing larger point estimates than BCE.

For practical DLQE scores, BCE showed a weak positive association (B = 1.923, 95% CI 0.144 to 3.702, *P* = 0.034), whereas PCE was not associated with practical scores. Detailed results for written, practical, and cognitive-domain scores are shown in Tables 4 and 5, including Standardised β coefficients for comparison of effect sizes.

**Table 4:** Associations between phase-based examinations and DLQE outcomes. Panel A. Associations with total written and practical DLQE scores

Examination	Written score B per 10-point increase (95% CI)	Standardised β	*P*	Practical score B per 10-point increase (95% CI)	Standardised β	*P*
BCE	14.355 (4.105 to 24.605)	0.207	0.006	1.923 (0.144 to 3.702)	0.174	0.034
PCE	18.455 (9.612 to 27.299)	0.295	< 0.001	0.607 (-0.928 to 2.141)	0.061	0.436

Analyses were restricted to graduates with complete BCE, PCE, graduation cohort, and DLQE outcome data (*n* = 172). Models included BCE and PCE scores, with graduation cohort included as a fixed factor. B values indicate the estimated change in DLQE outcome score associated with a 10-point increase in the corresponding phase-based examination score. Standardised β coefficients are reported to facilitate comparison of effect sizes across predictors

**Table 5:** Associations between phase-based examinations and DLQE outcomes. Panel B. Associations with cognitive-domain scores within the written DLQE

Examination	Memory-domain B per 10-point increase (95% CI)	Standardised β	*P*	Understanding-domain B per 10-point increase (95% CI)	Standardised β	*P*	Application-domain B per 10-point increase (95% CI)	Standardised β	*P*
BCE	3.377 (1.310 to 5.445)	0.133	0.002	5.456 (1.714 to 9.198)	0.159	0.005	5.521 (0.483 to 10.560)	0.156	0.032
PCE	2.557 (0.773 to 4.341)	0.112	0.005	7.721 (4.493 to 10.950)	0.250	< 0.001	8.177 (3.830 to 12.524)	0.256	< 0.001

Analyses were restricted to graduates with complete BCE, PCE, graduation cohort, and DLQE outcome data (*n* = 172). Models included BCE and PCE scores, with graduation cohort included as a fixed factor. B values indicate the estimated change in DLQE outcome score associated with a 10-point increase in the corresponding phase-based examination score. Standardised β coefficients are reported to facilitate comparison of effect sizes across predictors

### Internship institution type and DLQE outcomes

As a secondary institutional-level analysis, internship institution type was examined as a categorical indicator of clinical training context. In models adjusted for graduation cohort and the five course-cluster scores, none of the internship institution categories showed clear associations with written DLQE scores, practical DLQE scores, or written cognitive-domain scores, using other prefecture-level hospitals as the reference group. Detailed adjusted model results are provided in Supplementary Table S5.

### Model diagnostics

Model diagnostics did not identify highly influential observations. Maximum VIF values were below 5.0 in the primary course-cluster models and slightly above 5.0 in the restricted sensitivity models, suggesting at most moderate collinearity. Detailed diagnostic results for the primary course-cluster models are provided in Supplementary Table S6.

## Discussion

This single-institution retrospective cohort study found a domain-specific pattern in the associations between undergraduate academic assessment and DLQE outcomes in a five-year stomatology programme in China. Undergraduate academic indicators were more consistently associated with written DLQE scores and written cognitive-domain scores than with practical DLQE scores. Dental clinical course performance showed the clearest association with written DLQE outcomes, while phase-based examinations provided complementary information about written cognitive-domain performance. These findings suggest that DLQE outcomes may be useful external reference points for curriculum monitoring, but that written licensing outcomes and practical performance should be interpreted as related but distinct assessment domains.

The association between Dental clinical course performance and written DLQE outcomes may reflect the closer content overlap between undergraduate dental clinical courses and the written licensing examination. This finding is consistent with previous evidence that academic performance during dental training is associated with later academic or licensing-related outcomes [3, 12]. Interpreted through constructive alignment theory, the findings suggest that course-cluster performance may provide useful internal information about students’ preparation for written licensing examination domains when internal assessments and external examination outcomes address related content and cognitive demands [10]. However, these findings should not be interpreted as evidence that course-cluster scores comprehensively measure clinical competence or overall programme quality.

Basic science course performance showed positive associations with written DLQE scores and understanding-domain scores in the primary models, but these associations were not retained in the restricted sensitivity analyses. This pattern suggests that the Basic science findings were less robust than those for Dental clinical course performance. Although foundational biomedical knowledge remains important for dental education and clinical reasoning [15, 16], its independent association with DLQE outcomes in this study was weaker and more sensitive to cohort restriction. Therefore, Basic science course performance should be interpreted as a secondary and less stable finding.

Associations with practical DLQE scores were weaker than those with written outcomes. One possible explanation is that written course examinations and course-cluster scores mainly capture knowledge acquisition, knowledge integration, and written clinical reasoning [13, 16], whereas practical examination performance may also depend on procedural skill, communication, examination-day performance, and prior clinical exposure. This distinction is consistent with Miller’s framework of clinical competence, which differentiates knowledge-based assessment from assessment of performance in simulated or workplace settings [17]. Therefore, the weaker associations with practical DLQE scores may reflect differences in assessment format and competence level, rather than indicating that undergraduate education is unrelated to clinical skill development.

Although patient safety was not directly measured in this study, the distinction between written cognitive outcomes and practical performance has educational relevance. Dental practice requires not only knowledge of indications and principles, but also supervised development of procedural competence before patient care [18]. Accordingly, written academic assessment and written licensing outcomes should be considered alongside direct and observable evidence of hands-on clinical competence, such as practical examinations, simulation-based assessments, workplace-based assessments, direct observation, and structured feedback [9, 14, 17].

The phase-based examination findings suggest that BCE and PCE may provide complementary information about students’ preparation for written licensing examination outcomes. Both examinations were associated with written DLQE scores and with memory-domain, understanding-domain, and application-domain scores within the written examination. PCE showed larger point estimates than BCE for understanding-domain and application-domain scores, which may reflect its later timing and closer relevance to professional dental content. However, these analyses were secondary and were restricted to the 2021–2023 cohorts, corresponding to the period in which BCE and PCE data were available. BCE and PCE were introduced as institutional phase-based assessments to support staged academic monitoring and students’ progression from theoretical learning toward integrated professional knowledge and licensing-examination preparation. The present models evaluated associations between individual BCE/PCE scores and DLQE outcomes within the implementation cohorts. They cannot estimate the effect of introducing BCE/PCE or fully separate these associations from cohort-period or other time-related changes.

Undergraduate dental programmes are not designed solely around licensing examination performance. Their broader purpose is to support programme learning outcomes and prepare graduates for safe supervised practice and future professional development. International competency frameworks define graduate readiness through the integration of biomedical knowledge, clinical reasoning, procedural skills, professionalism, communication, ethical practice, patient-centred care, and preparedness for practice [19]. Within this broader framework, DLQE outcomes may serve as useful but incomplete external reference points. Domain-level DLQE feedback may help identify where internal academic assessments align with external written licensing requirements and where additional assessment evidence is needed, particularly for practical and workplace-based competence.

Internship institution type was not clearly associated with DLQE outcomes after adjustment for graduation cohort and course-cluster scores. This finding should not be interpreted as evidence that internship training is unimportant. Internship institution type was a broad institutional category and did not directly measure supervision quality, case mix, patient volume, procedural opportunities, feedback, workplace-based assessment, or student autonomy, which are more direct indicators of clinical learning experience and performance assessment [8, 9, 11]. In addition, internship placement may not have been random and may have been influenced by student characteristics, institutional assignment procedures, or student preferences. Therefore, the internship institution analysis should be interpreted as exploratory and not as evidence of causal effects of internship setting. Future multi-institutional and longitudinal studies should examine these more direct measures and their associations with written and practical licensing outcomes.

Exploratory differences by postgraduation pathway should also be interpreted cautiously. Postgraduation pathway may reflect prior academic performance, career selection, postgraduate examination preparation, and other unmeasured student characteristics rather than an independent educational effect. Therefore, these exploratory findings were not included in the main models and should be considered descriptive.

Several limitations should be acknowledged. First, this was a single-institution retrospective study, and the findings may not be generalisable to other dental schools, curricula, or licensing contexts. Second, DLQE outcomes were unavailable for 37 graduates, which may have introduced selection bias. Third, because of the staged DLQE structure, written and cognitive-domain outcomes were not applicable for graduates who did not pass the practical examination. Fourth, several potential baseline confounders were unavailable or incomplete in the analysed institutional dataset, including entrance examination scores, prior GPA, remediation history, and socioeconomic indicators. Age showed limited variability within this undergraduate cohort, and sex was not included in the present models. Residual confounding by unmeasured or incompletely measured baseline academic and demographic factors may therefore have influenced the observed associations. Fifth, BCE and PCE data were available only for the 2021–2023 cohorts after these institutional phase-based assessments were introduced. Therefore, the analyses were limited to score associations within the implementation cohorts and could not estimate the effect of introducing BCE/PCE or fully account for cohort-period or other time-related changes. Sixth, course grades, BCE, and PCE were subject to institutional assessment requirements and review procedures, but formal cross-course measurement standardisation, reliability indices, and item-level quality indicators were not included in the present analysis. Therefore, measurement variation across courses and examinations cannot be fully excluded. Seventh, cognitive-domain DLQE scores were written subdomain scores and should not be interpreted as direct measures of clinical performance. Eighth, internship institution type was a broad proxy for clinical training context and did not capture individual clinical exposure or supervision quality. Finally, multiple testing, moderate collinearity, residual outliers, and possible restricted range in practical scores should be considered when interpreting weaker or borderline associations, although model diagnostics did not identify highly influential observations.

Overall, the findings are consistent with a domain-specific interpretation of assessment alignment in undergraduate dental education. Dental clinical course performance was the undergraduate academic indicator most consistently associated with written DLQE outcomes, while BCE and PCE provided complementary information about written cognitive-domain performance. Associations with practical DLQE scores were weaker, suggesting that written academic assessments and written licensing outcomes should be complemented by direct evidence of procedural and workplace-based competence. DLQE outcomes may therefore be useful external reference points for curriculum monitoring, but they should be interpreted as incomplete indicators of graduate readiness within the broader goals of undergraduate dental education.

## Conclusions

Undergraduate academic assessment showed domain-specific associations with DLQE outcomes in this single-institution cohort. Dental clinical course performance was most consistently associated with written DLQE scores and written cognitive-domain scores, whereas associations with practical DLQE scores were weaker and less consistent. BCE and PCE provided complementary information about written DLQE performance, but these secondary findings should be interpreted cautiously because phase-based examination data were limited to the 2021–2023 cohorts. Internship institution type showed no clear independent association with DLQE outcomes after adjustment. These findings suggest that DLQE outcomes may serve as useful external reference points for curriculum monitoring and assessment alignment, but they should be interpreted as incomplete indicators of graduate readiness and considered alongside direct evidence of clinical performance and workplace-based competence.

## Supplementary Information

Supplementary Material 1.

## Data Availability

The datasets generated and analysed during the current study are not publicly available because they contain institutional educational records and are subject to institutional data governance restrictions, but anonymized data may be available from the corresponding author on reasonable request and with permission from Zhejiang Chinese Medical University.”

## Abbreviations

BCE: Basic Comprehensive Examination
CI: Confidence interval
DLQE: Dental Licensing Qualification Examination
PCE: Professional Comprehensive Examination
SD: Standard deviation
UDE: Undergraduate dental education
ZCMU: Zhejiang Chinese Medical University
ZJU: Zhejiang University
