# Supplementary material for: Undergraduate academic assessment and dental licensing examination outcomes: a single-institution retrospective cohort study in China
Source: BMC Med Educ. 2026 Jul 18;26:1260. doi: 10.1186/s12909-026-09971-5 (PMC13445858; doi:10.1186/s12909-026-09971-5)
Supplement: Supplementary file 1 [file 12909_2026_9971_MOESM1_ESM.docx]

**Supplementary Table S1. Characteristics of the overall analysed cohort (n = 278) and the 2021–2023 restricted cohort (n = 172)**

| Characteristic | Overall analysed cohort | 2021–2023 restricted cohort |
| --- | --- | --- |
| **Sample size** | 278 | 172 |
| **Graduation cohort, n (%)** |  |  |
| 2019 | 53 (19.1) | 0 (0.0) |
| 2020 | 53 (19.1) | 0 (0.0) |
| 2021 | 59 (21.2) | 59 (34.3) |
| 2022 | 60 (21.6) | 60 (34.9) |
| 2023 | 53 (19.1) | 53 (30.8) |
| **Credit-weighted mean course-cluster scores, mean ± SD** |  |  |
| Basic science | 79.73 ± 6.36 | 79.45 ± 6.52 |
| Dental clinical | 84.20 ± 5.44 | 83.27 ± 5.08 |
| General clinical | 74.85 ± 6.33 | 74.50 ± 6.48 |
| Medical humanities | 81.28 ± 7.06 | 81.96 ± 7.70 |
| Preventive medicine | 81.86 ± 8.37 | 83.29 ± 8.05 |
| **DLQE outcomes, mean ± SD** |  |  |
| Practical score | 78.06 ± 6.95 | 78.36 ± 6.55 |
| Written score | 410.58 ± 40.93 | 408.56 ± 41.23 |
| Memory-domain score | 58.80 ± 18.01 | 68.43 ± 15.10 |
| Understanding-domain score | 150.88 ± 20.72 | 144.80 ± 20.37 |
| Application-domain score | 200.89 ± 22.93 | 195.33 ± 21.04 |
| **First-attempt DLQE result, n (%)** |  |  |
| Passed | 245 (88.1) | 151 (87.8) |
| Not passed | 33 (11.9) | 21 (12.2) |
| **Internship institution type, n (%)** |  |  |
| ZJU affiliated hospitals | 124 (44.6) | 86 (50.0) |
| ZCMU affiliated hospitals | 96 (34.5) | 55 (32.0) |
| Other hospitals in Hangzhou | 36 (12.9) | 20 (11.6) |
| Other prefecture-level hospitals | 22 (7.9) | 11 (6.4) |

Values are presented as mean ± SD or n (%). Course-cluster scores were calculated as credit-weighted mean final course scores on the original 0–100 scale. The 2021–2023 restricted cohort corresponded to the cohorts in which BCE and PCE data were available and was used for restricted sensitivity analyses. Practical-score analyses and first-attempt pass-rate calculations included all graduates with available practical examination scores. In the overall analysed cohort, written-score and cognitive-domain outcomes were available for 276 graduates because two graduates were not eligible for the written examination after not passing the practical examination. In the 2021–2023 restricted cohort, written-score and cognitive-domain outcomes were available for all 172 graduates.

**Supplementary Table S2. Mapping between undergraduate course clusters and DLQE written subjects**

| Undergraduate course cluster used in main analysis | Undergraduate curriculum component | Corresponding DLQE written subject | DLQE written subject domain | Mapping status |
| --- | --- | --- | --- | --- |
| Dental clinical | Pediatric Dentistry | Pediatric Dentistry | Dental clinical subjects | Direct counterpart |
|  | Oral and Maxillofacial Surgery | Oral and Maxillofacial Surgery |  |  |
|  | Oral and Maxillofacial Radiology | Oral and Maxillofacial Radiology |  |  |
|  | Oral Mucosal Diseases | Oral Mucosal Diseases |  |  |
|  | Prosthodontics | Prosthodontics |  |  |
|  | Operative Dentistry and Endodontics | Operative Dentistry and Endodontics |  |  |
|  | Periodontology | Periodontology |  |  |
| Basic science | Oral Anatomy and Physiology | Oral Anatomy and Physiology | Basic science |  |
|  | Oral Histopathology | Oral Histopathology |  |  |
|  | Medical Microbiology | Medical Microbiology |  |  |
|  | Medical Immunology | Medical Immunology |  |  |
|  | Biochemistry | Biochemistry |  |  |
|  | Pharmacology | Pharmacology |  |  |
| General clinical | Internal Medicine | Internal Medicine | General clinical medicine |  |
|  | Surgery | Surgery |  |  |
| Not included in course cluster scores | **Elective clinical course with very low enrolment** | Gynecology |  | No undergraduate counterpart |
|  | **Elective clinical course with very low enrolment** | Pediatrics |  |  |
| Medical humanities | Medical Psychology | Medical Psychology | Medical humanities | Direct counterpart |
|  | Medical Ethics and Health Law | Medical Ethics |  | Covered in integrated course |
|  | Medical Ethics and Health Law | Health Law |  |  |
| Preventive medicine | Preventive Medicine | Preventive Medicine | Preventive medicine | Direct counterpart |
|  | Preventive Dentistry | Preventive Dentistry |  |  |

DLQE, Dental Licensing Qualification Examination. This table shows how undergraduate curriculum components were mapped to DLQE written subjects and then grouped into the course clusters used in the main analysis. Medical Ethics and Health Law was taught as an integrated undergraduate course and mapped to two DLQE written subjects. Gynecology and Pediatrics were offered as elective clinical courses with very low enrolment and therefore were not included in the course-cluster scores because comparable cohort-level course-grade data were not available.

**Supplementary Table S3. Exploratory comparison of DLQE outcomes by postgraduation pathway**

| DLQE outcome | Neither employment nor postgraduate study  mean ± SD (n = 9) | Employment  mean ± SD (n = 183) | Postgraduate study  mean ± SD (n = 86) | Overall *P* |
| --- | --- | --- | --- | --- |
| Practical DLQE score | 72.22 ± 5.14 | 77.67 ± 6.91 | 79.51 ± 6.84 | 0.004 |
| Written DLQE score | 390.67 ± 45.85 | 401.06 ± 41.78 | 433.07 ± 27.81 | <0.001 |
| Memory | 61.57 ± 14.87 | 57.58 ± 18.00 | 61.12 ± 18.26 | 0.293 |
| Understanding | 143.67 ± 26.33 | 146.88 ± 20.43 | 160.23 ± 17.66 | <0.001 |
| Application | 185.42 ± 23.92 | 196.61 ± 23.39 | 211.72 ± 17.42 | <0.001 |

Values are presented as mean ± SD. P values were derived from one-way ANOVA. These analyses were exploratory and were not included in the main models. Written-score and cognitive-domain analyses used graduates with available written examination results.

**Supplementary Table S4. Sensitivity analyses of course-cluster scores restricted to the 2021–2023 cohorts**

| **Panel A. Total written and practical DLQE scores** | | | |  |
| --- | --- | --- | --- | --- |
| Course cluster | Written score B per 10-point increase (95% CI) | *P* | Practical score B per 10-point increase (95% CI_ | *P* |
| Basic science | 4.371 (-10.782 to 19.525) | 0.570 | -1.437 ( -4.461 to 1.586) | 0.349 |
| Dental clinical | 33.623 (16.672 to 50.575) | <0.001 | 2.173 (-1.209 to 5.556) | 0.206 |
| General clinical | 4.211 (-7.941 to 16.364) | 0.495 | 2.023 (-0.402 to 4.448) | 0.101 |
| Medical humanities | 0.711 (-13.175 to 14.598) | 0.920 | 2.830 (0.059 to 5.601) | 0.045 |
| Preventive medicine | 7.760 (-2.840 to 18.360) | 0.150 | -0.975 (-3.090 to 1.140) | 0.364 |

| **Panel B. Cognitive-domain scores within the written DLQE** | | | | | | |
| --- | --- | --- | --- | --- | --- | --- |
| Course cluster | Memory-domain B (95% CI) | *P* | Understanding-domain B (95% CI) | *P* | Application-domain B (95% CI) | *P* |
| Basic science | 0.884 (-2.276 to 4.044) | 0.581 | 3.367 (-2.346 to 9.081) | 0.246 | 0.120 (-7.401 to 7.641) | 0.975 |
| Dental clinical | 4.948 (1.414 to 8.483) | 0.006 | 11.356 (4.965 to 17.748) | 0.001 | 17.319 (8.905 to 25.733) | <0.001 |
| General clinical | 0.808 (-1.726 to 3.342) | 0.530 | 1.344 (-3.238 to 5.926) | 0.563 | 2.059 (-3.973 to 8.090) | 0.501 |
| Medical humanities | 1.801 (-1.094 to 4.697) | 0.221 | -0.320 (-5.556 to 4.916) | 0.904 | -0.770 (-7.662 to 6.123) | 0.826 |
| Preventive medicine | 0.998 (-1.213 to 3.208) | 0.374 | 2.332 (-1.665 to 6.329) | 0.251 | 4.431 (-0.831 to 9.692) | 0.098 |

Analyses were restricted to graduates from the 2021–2023 cohorts, corresponding to the period in which BCE and PCE data were available. Models included the five credit-weighted mean course-cluster scores simultaneously, with graduation cohort included as a fixed factor; the 2023 cohort served as the reference group. B values indicate the estimated change in DLQE outcome score associated with a 10-point increase in the corresponding credit-weighted mean course-cluster score. In model diagnostics, maximum Cook’s distance values ranged from 0.052 to 0.190 across models, and the maximum VIF was 5.203, suggesting no substantial influential-case problem and moderate but not severe collinearity.

**Supplementary Table S5. Adjusted associations between internship institution type and DLQE outcomes**

| Internship institution type | Written score B (95% CI) | *P* | Practical score B (95% CI) | *P* | Memory-domain B (95% CI) | *P* | Understanding-domain B (95% CI) | *P* | Application-domain B (95% CI) | *P* |
| --- | --- | --- | --- | --- | --- | --- | --- | --- | --- | --- |
| ZJU affiliated hospitals | -8.804  (-23.728 to 6.120) | 0.246 | -0.376  (-3.438 to 2.686) | 0.809 | -1.652  (-4.508 to 1.203) | 0.256 | -3.239  (-9.148 to 2.670) | 0.281 | -3.912  (-11.403 to 3.578) | 0.305 |
| ZCMU affiliated hospitals | -6.637  (-21.789 to 8.515) | 0.389 | -2.150  (-5.254 to 0.953) | 0.174 | -1.530  (-4.429 to 1.369) | 0.300 | -2.634  (-8.633 to 3.365) | 0.388 | -2.473  (-10.078 to 5.132) | 0.523 |
| Other hospitals in Hangzhou | -4.610  (-21.970 to 12.750) | 0.601 | -0.578  (-4.153 to 2.998) | 0.751 | -0.888  (-4.209 to 2.434) | 0.599 | -1.831  (-8.703 to 5.042) | 0.600 | -1.892  (-10.605 to 6.821) | 0.669 |

Models included internship institution type, the five credit-weighted mean course-cluster scores, and graduation cohort fixed effects. Other prefecture-level hospitals served as the reference group. Practical-score analyses included all graduates with available practical examination scores (n = 278), whereas written-score and cognitive-domain analyses included graduates with available written examination results (n = 276). Course-cluster and graduation cohort coefficients are not shown.

**Supplementary Table S6. Diagnostics for the primary course-cluster regression models shown in Table 2**

| Model | n | Adjusted R² | Maximum VIF | Standardized residual range | Maximum Cook’s distance |
| --- | --- | --- | --- | --- | --- |
| Written score | 276 | 0.408 | 4.089 | -3.251 to 2.720 | 0.066 |
| Practical score | 278 | 0.096 | 4.108 | -2.992 to 2.423 | 0.039 |
| Memory-domain score | 276 | 0.888 | 4.089 | -4.418 to 2.524 | 0.102 |
| Understanding-domain score | 276 | 0.639 | 4.089 | -3.092 to 2.923 | 0.086 |
| Application-domain score | 276 | 0.525 | 4.089 | -3.024 to 2.337 | 0.054 |

VIF, variance inflation factor. Models included the five credit-weighted mean course-cluster scores and graduation cohort fixed effects. Practical-score analyses included all graduates with available practical examination scores (n = 278), whereas written-score and cognitive-domain analyses included graduates with available written examination results (n = 276). Standardized residual ranges and Cook’s distance values were obtained from linear regression diagnostics. No Cook’s distance exceeded 1.0, and maximum VIF values were below 5.0.
